# P-771. Trends in Urinary Antimicrobial Resistance Among Veterans Affairs Spinal Cord Injury Patients — United States, 1999–2023

**DOI:** 10.1093/ofid/ofaf695.982

**Published:** 2026-01-11

**Authors:** Guillermo Rodriguez-Nava, Vanessa El Kamari, Jorge Salinas, John Lavelle, John Hornberger, Cybele Renault

**Affiliations:** Stanford University School of Medicine, Stanford, California; Stanford University, Stanford, California; Stanford University, Stanford, California; Veterans Affairs Palo Alto Health Care System, Palo Alto, California; Veterans Affairs Palo Alto Health Care System, Palo Alto, California; Veterans Affairs Palo Alto Health Care System, Palo Alto, California

## Abstract

**Background:**

Identifying evolving antimicrobial resistance patterns is essential for public health, stewardship, and infection control. The veteran spinal cord injury (SCI) population is particularly vulnerable, facing antibiotic pressure from recurrent urinary tract infections and treatment of asymptomatic bacteriuria— both drivers of resistance. We characterized 24-year temporal and regional trends in antimicrobial resistance among urinary pathogens in SCI patients across the U.S. Veterans Affairs.

**Figure:**
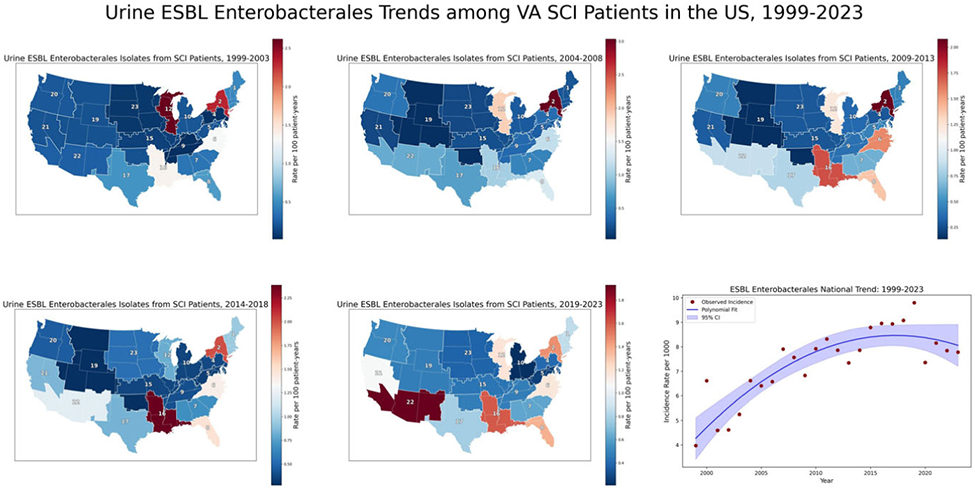
Temporal and Regional Trends in ESBL-Producing Enterobacterales Among SCI Patients — United States, 1999–2023

VISN-level incidence rates of Enterobacterales with an ESBL phenotype are expressed per 100 person-years and displayed as 5-year choropleth maps. National incidence rates per 1,000 population are shown as an annual trend line smoothed using second-degree polynomial regression. A clockwise geographic spread was observed, originating in the Midwest and Northeast and extending to the South and Southwest, with persistently elevated rates along this trajectory. National rates increased through 2015 before plateauing.

**Figure:**
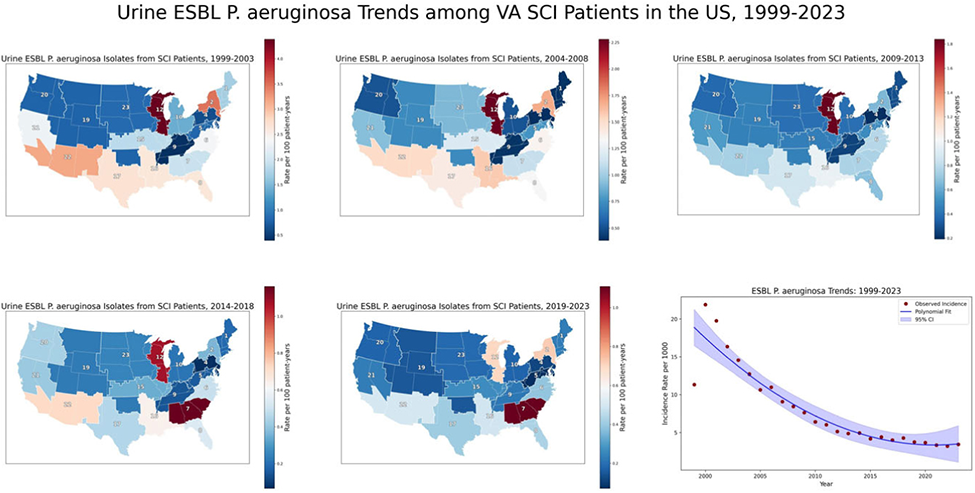
Temporal and Regional Trends in ESBL-Producing Pseudomonas aeruginosa Among SCI Patients — United States, 1999–2023

VISN-level incidence rates of Pseudomonas aeruginosa with an ESBL phenotype are expressed per 100 person-years and displayed as 5-year choropleth maps. National incidence rates per 1,000 population are shown as an annual trend line smoothed using second-degree polynomial regression. Unlike Enterobacterales, no clear geographic shift was observed; however, resistance remained widespread, with relatively higher incidence in the Midwest, Northeast, South, and Southwest. National rates steadily declined over time.

**Methods:**

We conducted a retrospective cohort study of urine cultures from SCI patients across 1,380 VA facilities from 1999 to 2023. Positive cultures for Enterobacterales (*Escherichia coli* and *Klebsiella pneumoniae*) and *Pseudomonas aeruginosa* were identified. Extended-spectrum beta-lactamase (ESBL) phenotype and carbapenem resistance (CR) were defined according to Clinical and Laboratory Standards Institute susceptibility criteria. Incidence rates were calculated per 100 person-years, stratified by Veterans Integrated Service Networks (VISNs) in 5-year intervals, and visualized using choropleth maps. National trends were modeled using second-degree polynomial regression to capture non-linear incidence patterns.

**Figure:**
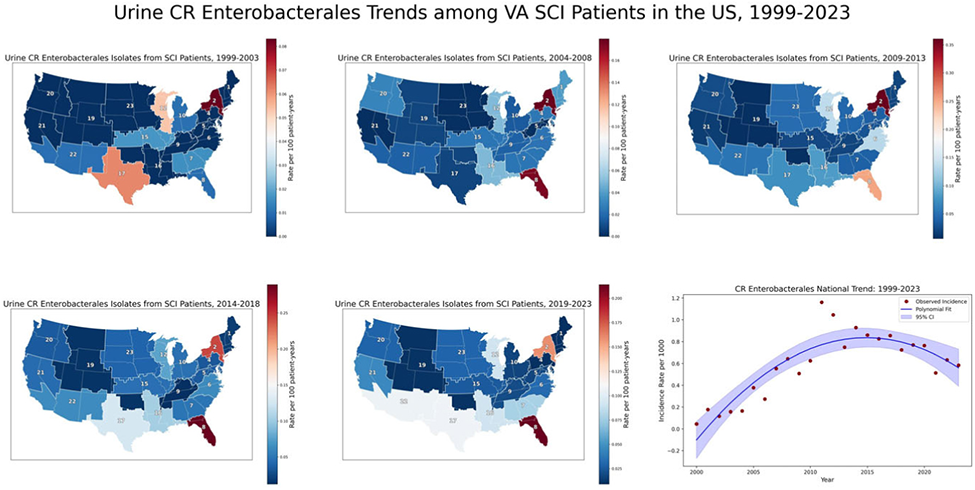
Temporal and Regional Trends in Carbapenem-Resistant Enterobacterales Among SCI Patients — United States, 1999–2023

VISN-level incidence rates of carbapenem-resistant Enterobacterales are expressed per 100 person-years and displayed as 5-year choropleth maps. National incidence rates per 1,000 population are shown as an annual trend line smoothed using second-degree polynomial regression. Resistance demonstrated a clockwise geographic shift, with the South and Southwest—particularly VISNs 8, 16, 17, and 22—emerging as recent hotspots. The national trend peaked around 2015 before beginning to decline.

**Figure:**
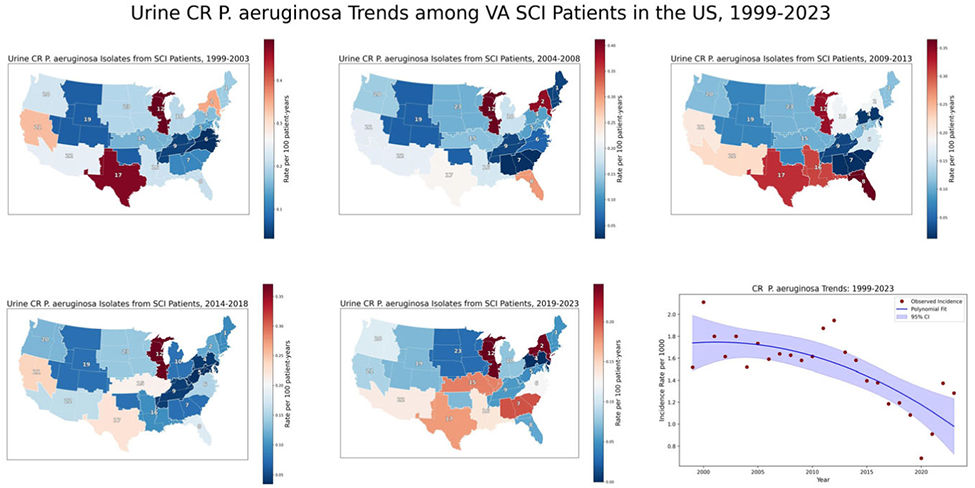
Temporal and Regional Trends in Carbapenem-Resistant Pseudomonas aeruginosa Among SCI Patients — United States, 1999–2023

VISN-level incidence rates of carbapenem-resistant Pseudomonas are expressed per 100 person-years and displayed as 5-year choropleth maps. National incidence rates per 1,000 population are shown as an annual trend line smoothed using second-degree polynomial regression. Resistance was widespread, with greater density in the Midwest, Northeast, and South; VISN 12 remained consistently elevated across all intervals. The national trend showed a gradual decline over time.

**Results:**

We identified 49,326 SCI patients who contributed 302,495 unique urine cultures over 24 years. Resistance patterns evolved more consistently by organism than by phenotype. Enterobacterales showed increasing national incidence through 2015 followed by plateauing, with a clockwise geographic spread beginning in the Midwest and Northeast, extending to the South and Southwest, and leaving a lasting pattern of elevated resistance along the way. In contrast, *P. aeruginosa* showed a steady national decline in resistance, with no clear geographic shift but widespread distribution and persistently higher incidence in the Midwest, Northeast, South, and Southwest. VISN 12 was an early and persistent hotspot for resistance.

**Conclusion:**

*Enterobacterales* showed rising resistance with a notable geographic shift toward the South and Southwest. In contrast, *P. aeruginosa* demonstrated continued national declines persistently widespread distribution. These shifts may reflect stewardship efforts, patient movement, or other external pressures.

**Disclosures:**

All Authors: No reported disclosures

